# The susceptibility of SERPINE1 rs1799889 SNP in diabetic vascular complications: a meta-analysis of fifty-one case-control studies

**DOI:** 10.1186/s12902-021-00837-z

**Published:** 2021-09-30

**Authors:** JingYi Chen, ChuanNan Zhai, ZhiQian Wang, Rui Li, WenJing Wu, Kai Hou, Mohammad Alzogool, Yan Wang, HongLiang Cong

**Affiliations:** 1 School of Medicine, NanKai University, Weijin Road No. 94, Nankai District, 300071 Tianjin, China; 2 Tianjin Key Lab of Ophthalmology and Visual Science, Tianjin Eye Institute, Tianjin Eye Hospital, Gansu Road No. 4, Heping District, 300020 Tianjin, China; 3 Department of Cardiology, Tianjin Chest Hospital, Taierzhuang south Road No. 291, Jinnan District, 300350 Tianjin, China; 4 Department of Optometry, Shenyang Eye Institute, The 4th People’s Hospital of Shenyang, No 20. Huanghe South Avenue, Huanggu District, 110031 Shenyang, Liaoning China; 5 Tianjin GongAn Hospital, Nanjing Road No. 78, Heping District, 300042 Tianjin, China

**Keywords:** SERPINE1, rs1799889, 4G/5G polymorphism, Plasminogen activator inhibitor 1, Diabetes, Diabetic vascular disease

## Abstract

**Background:**

The serine protease inhibitor-1 (SERPINE1) rs1799889 single nucleotide polymorphism (SNP) has been constantly associated with diabetes mellitus (DM) and its vascular complications. The aim of this meta-analysis was to evaluate this association with combined evidences.

**Methods:**

The systematic search was performed for studies published up to March 2021 which assess the associations between SERPINE1 rs1799889 SNP and the risks of DM, diabetic retinopathy (DR), diabetic cardiovascular disease (CVD) and diabetic nephropathy (DN). Only case-control studies were identified, and the linkage between SERPINE1 rs1799889 polymorphism and diabetic vascular risks were evaluated using genetic models.

**Results:**

51 comparisons were enrolled. The results revealed a significant association with diabetes risk in overall population (allelic: OR = 1.34, 95 % CI = 1.14–1.57, homozygous: OR = 1.66, 95 % CI = 1.23–2.14, heterozygous: OR = 1.35, 95 % CI = 1.08–1.69, dominant: OR = 1.49, 95 % CI = 1.18–1.88, recessive: OR = 1.30, 95 % CI = 1.06–1.59) as well as in Asian descents (allelic: OR = 1.45, 95 % CI = 1.16–1.82, homozygous: OR = 1.88, 95 % CI = 1.29–2.75, heterozygous: OR = 1.47, 95 % CI = 1.08-2.00, dominant: OR = 1.64, 95 % CI = 1.21–2.24, recessive: OR = 1.46, 95 % CI = 1.09–1.96). A significant association was observed with DR risk (homozygous: OR = 1.25, 95 % CI = 1.01–1.56, recessive: OR = 1.20, 95 % CI = 1.01–1.43) for overall population, as for the European subgroup (homozygous: OR = 1.32, 95 % CI = 1.02–1.72, recessive: OR = 1.38, 95 % CI = 1.11–1.71). A significant association were shown with DN risk for overall population (allelic: OR = 1.48, 95 % CI = 1.15–1.90, homozygous: OR = 1.92, 95 % CI = 1.26–2.95, dominant: OR = 1.41, 95 % CI = 1.01–1.97, recessive: OR = 1.78, 95 % CI = 1.27–2.51) and for Asian subgroup (allelic: OR = 1.70, 95 % CI = 1.17–2.47, homozygous: OR = 2.46, 95 % CI = 1.30–4.66, recessive: OR = 2.24, 95 % CI = 1.40–3.59) after ethnicity stratification. No obvious association was implied with overall diabetic CVD risk in any genetic models, or after ethnicity stratification.

**Conclusions:**

SERPINE1 rs1799889 4G polymorphism may outstand for serving as a genetic synergistic factor in overall DM and DN populations, positively for individuals with Asian descent. The association of SERPINE1 rs1799889 SNP and DR or diabetic CVD risks was not revealed.

**Supplementary Information:**

The online version contains supplementary material available at 10.1186/s12902-021-00837-z.

## Background

Diabetes mellitus (DM) is a major worldwide epidemic that has gained significant public attention. According to recent data from the latest WHO report on diabetes, its world prevalence has been estimated at 8.4 % [1]. Added to this universal health issue, patients with diabetes often develop several vascular and neurogenic complications such as nephropathy, coronary heart disease, myocardial infarction, ischemic stroke, retinopathy, and neuropathy [2]. Most diabetic patients suffer from at least one complication, and vascular complications have become the leading cause of morbidity and mortality, while neurogenic complications such as retinopathy can severely affect quality of life [3].

To date, advances in epidemiological and pathophysiological research on DM have improved our understanding of the underlying pathogenic mechanism of diabetes. The determinants of DM consist of a matrix of genetic susceptibility and epigenetic and lifestyle factors that interact with one another and operate within the larger physical-sociocultural environment [2, 4]. Genetic elements are essentially involved in the pathogenesis of diabetes [5]. Plasminogen activator inhibitor 1 (PAI-1) belongs to the serine protease inhibitor (SERPINE) superfamily and plays a substantial role in the modulation of fibrinolysis and thrombosis [6]. The SERPINE1 gene is commonly recognized in the literature as PAI-1 gene and has been widely studied in epidemiologic studies. A common promoter SNP- rs1799889 consists in an A > G substitution located 2KB upstream the SERPINE1 gene. The 4G allele in the promoter region at nucleotide position-675 is associated with higher PAI-1 levels compared to the 5G allele [7]. PAI-1 levels increase in the pre-diabetic as well as the diabetic state [8]. Moreover, increases in PAI-1 expression may contribute to vascular complications such as nephropathy, coronary heart disease, myocardial infarction, and ischemic stroke [8, 9].

To date, there have been extensive studies conducted investigating the potential role of SERPINE1 rs1799889 polymorphism in DM and subsequent complications. However, former meta-analyses reached inconsistent conclusions on this topic as they might be restrained by sample sizes or an insufficiency of studies [10, 11]. Contradictory as the previous results might be, recent investigations by Li et al. [12] and Xu et al. [13] defined the SERPINE1 rs1799889 SNP genotype dominant allele model as a risk factor for vascular complications in patients with DM. As a result, we felt obliged to perform the updated meta-analysis with larger sample sizes and more sufficient data, intending to better solve the disparity and further evaluate the associations between SERPINE1 rs1799889 SNP polymorphism and DM vascular complications.

## Method

### Search strategy

The current meta-analysis was performed according to the Preferred Reporting Items for Systematic Reviews and Meta-Analyses (PRISMA) statement [14]. Potentially related articles were systematically searched in PubMed, Medline, Embase, CNKI, OVID, ScienceDirect and WanFang to identify published literatures up to March 2021 using the following key words: “diabetes mellitus (DM)”, “diabetes”, “diabetic”, and “plasminogen activator inhibitor-1”, “PAI-1”, “PAI 1”, “SERPINE1”, “polymorphism, genetic”, “polymorphism, single-stranded conformational”, “polymorphism, single nucleotide”, “polymorphism, restriction fragment length”, “variants”, “variations, DNA copy number”, “genotype”, “allele”, “mutation”, “mutation, frameshift”, “INDEL mutation”, “rs1799889”, “4G”, “5G”, “4G/5G”, and “diabetes complications”, “coronary artery/heart disease (CAD/CHD)”, “cardiovascular disease (CVD)”, “myocardial infarction”, “ischemic heart disease”, “ischemic stroke”, or “nephropathy”, “renal disease”, or “retinopathy”, “diabetic retinopathy”, “retinal artery occlusion”. No language restrictions were imposed in this meta-analysis. Furthermore, the reference lists of all retrieved articles were screened to identify potentially relevant studies. The literature search was independently performed by two reviewers (JY Chen and CN Zhai).

### Inclusion and exclusion criteria

A study included in this meta-analysis must meet with the following criteria: (1) case-control study on correlation analysis between SERPINE1 rs1799889 SNP and the risk of diabetes and associated complications to be assessed; (2) the study must include original and adequate data to allow calculation of odds ratios (ORs) with 95 % confidence intervals (CIs) (independence among studies); (3) evaluation of SERPINE1 rs1799889 polymorphism and the risk of diabetes and its complications.

A study was excluded when fulfilling one of the following criteria: (1) for overlapping-data study, only the most recent and complete one was enrolled; (2) study with missing information (particularly genotype distributions), while the required information could not be acquired from the corresponding author; (3) genome scans investigating linkages with no detailed genotype frequencies between cases and controls. If inclusions have disagreements, we reached a consensus through discussion. Two reviewers (JY Chen and CN Zhai) independently screened the titles and abstracts for the eligibility criteria. Subsequently, reviewers both read the full text of the studies which potentially met with the inclusion criteria, and the literature was reviewed to determine final inclusive data.

### Data extraction

Two reviewers (JY Chen and CN Zhai) conducted the data extraction from each study independently. Any disagreement between the two reviewers was solved by discussion with the third reviewer (ZQ Wang) until reaching a consensus. Three reviewers (JY Chen, CN Zhai, and ZQ Wang) independently evaluated the quality of each case-control study by using the Newcastle–Ottawa Scale criteria [15]. We summarized the information extracted from each literature in Table 1. The characteristics of the selected studies included (1) name of first author; (2) year of publication; (3) country in which the study was done; (4) ethnicity; (5) the number of cases and controls; (6) the genotypic distributions of SERPINE1 rs1799889 polymorphisms in cases and controls; (7) type of disease and outcome. Furthermore, the probability value (*P* value) of Hardy-Weinberg equilibrium (HWE) test was also calculated on the basis of allele frequencies of certain SERPINE1 rs1799889 polymorphisms in the control group.

**Table 1:** Characteristics and genotype frequencies for the SERPINE1 rs1799889 SNP in the included studies

Study	Year	Country	Ethnicity	Sample size Case/Control		Study type	Outcomes	Genotyping methods	5G allele frequency		HWE
									Case/Control (%)		
Mansfield et al	1995	UK	European	38	122	Hospital based	CAD & NIDDM	PCR	27.6	42.2	Y
Nagi et al	1997	USA	Mix	70	101	Population based	DR & NIDDM	PCR	48.6	60.3	Y
Broch et al	1998	Spain	European	82	95	Hospital based	DR & NIDDM	PCR	51.2	54.7	Y
Kimura et al	1998	Japan	Asian	208	177	Population based	NIDDM	PCR	41.3	40.1	Y
				110	98	Population based	PDR & NIDDM	PCR	42.7	39.8	Y
				110	98	Population based	DN & NIDDM	PCR	41.8	40.9	Y
De Cosmo et al	1999	Italy & UK	European	311	200	Population based	IDDM	PCR	48.6	49.0	Y
				175	136	Population based	DN & IDDM	PCR	47.1	50.4	Y
Wong et a	2000	Hong Kong	Asian	84	57	Hospital based	DR & NIDDM	PCR	40.5	47.4	Y
				95	46	Hospital based	DN & NIDDM	PCR	39.5	51.1	Y
Tarnow et al	2000	Denmark	European	197	191	Hospital based	DN & IDDM	PCR	46.2	46.1	Y
Ding et al	2001	China	Asian	112	169	Hospital based	NIDDM	PCR	56.3	67.2	Y
				49	63	Hospital based	CHD & NIDDM	PCR	54.9	64.3	Y
Li et al	2001	China	Asian	143	85	Hospital based	NIDDM	PCR	41.3	44.7	Y
				79	64	Hospital based	DN & NIDDM	PCR	39.2	43.8	Y
Petrovic et al	2003	Slovenia	European	154	194	Population based	MI & NIDDM	PCR	46.8	42.0	Y
Santos et al	2003	Brazil	European	99	111	Hospital based	DR & NIDDM	PCR	55.1	53.6	Y
Globocnik-P et al	2003	Slovenia	European	124	80	Hospital based	DR & NIDDM	PCR	45.2	43.8	Y
Lopes et al	2003	France	European	229	406	Population based	CHD & NIDDM	PCR	44.1	48.9	Y
Liu et al	2004	China	Asian	147	26	Hospital based	NIDDM	PCR	45.9	53.8	Y
				56	91	Hospital based	DR & NIDDM	PCR	50.0	43.4	Y
				77	70	Hospital based	DN & NIDDM	PCR	42.9	49.3	Y
Pan et al	2004	China	Asian	204	60	Hospital based	NIDDM	PCR	52.7	56.7	Y
Li et al	2004	China	Asian	54	54	Population based	NIDDM	PCR	42.6	46.3	Y
Murata et al	2004	Japan	Asian	188	92	Hospital based	DR & NIDDM	PCR	35.6	34.2	Y
Tang et al	2004	China	Asian	108	38	Hospital based	NIDDM	PCR	38.9	46.1	Y
				59	49		DN & NIDDM	PCR	31.4	48.0	Y
Wang et al	2004	China	Asian	114	30	Hospital based	NIDDM	PCR	34.6	61.7	Y
				76	38	Hospital based	DN & NIDDM	PCR	28.3	47.4	Y
Meigs et al	2006	USA	European	216	1953	Population based	DM	PCR	46.1	47.4	Y
Zietz et al	2006	Germany	European	192	312	Population based	DR & NIDDM	PCR	42.4	44.4	Y
				189	320	Population based	CHD & NIDDM	PCR	45.8	42.7	Y
Martin et al	2007	Ireland	European	222	361	Hospital based	DN & IDDM	PCR	42.8	44.5	Y
Zheng et al	2007	China	Asian	247	87	Hospital based	NIDDM	PCR	44.3	46.0	Y
				167	80	Hospital based	DN & NIDDM	PCR	40.7	51.9	Y
Saely et al	2008	Austria	European	148	524	Population based	NIDDM	PCR	43.9	47.6	Y
Yan et al 1	2008	China	Asian	66	33	Hospital based	NIDDM	PCR	50.8	56.1	Y
Yan et al 2	2008	China	Asian	217	58	Population based	NIDDM	PCR	53.9	79.3	Y
				125	92	Population based	DN & NIDDM	PCR	42.4	69.6	Y
Ezzidi et al	2009	Tunisia	European	383	473	Hospital based	DR & NIDDM	PCR	58.1	63.0	Y
Prasad et al	2010	India	Mix	196	225	Hospital based	DN & NIDDM	PCR	48.0	50.9	Y
Xue et al	2010	China	Asian	120	50	Hospital based	NIDDM	PCR	41.7	70.0	Y
				70	50	Hospital based	DN & NIDDM	PCR	20.7	71.0	Y
Liu et al	2011	China	Asian	63	39	Hospital based	NIDDM	PCR	39.7	57.7	Y
				29	34	Hospital based	DN & NIDDM	PCR	44.8	35.3	Y
Tan et al	2011	China	Asian	30	50	Hospital based	CHD & NIDDM	PCR	35.0	48.0	Y
Al-Hamodi et al	2012	Malaysia	Asian	303	131	Population based	NIDDM	PCR	50.0	53.1	Y
Weng et al	2012	Taiwan	Asian	27	251	Hospital based	PTDM	PCR	53.7	40.0	Y
Xu et al	2016	China	Asian	107	101	Hospital based	NIDDM	PCR	37.9	47.0	Y
				65	42	Hospital based	DN & NIDDM	PCR	37.7	38.1	Y
Li et al	2018	China	Asian	175	125	Hospital based	IS & NIDDM	PCR	42.6	36.8	Y

*CAD* coronary artery disease, *CHD* coronary heart disease, *MI* myocardial infarction, *IS* ischemic stroke, *IDDM* insulin-dependent diabetes mellitus, *NIDDM* non-insulin-dependent diabetes mellitus, *PTDM* post-transplant diabetes mellitus, *PCR* polymerase chain reaction, *HWE* Hardy-Weinberg equilibrium, *Y* Yes

### Statistical analysis

All statistical analyses were conducted using STATA 12.0 (Stata-corp, college station, Tex) and Review Manager Version 5.3.3 (The Cochrane Collaboration, Software Update, Oxford, United Kingdom). The associations between the SERPINE1 rs1799889 polymorphism and DM and its complications’ susceptibility were assessed using the following genetic models: 4G vs. 5G (allelic), 4G4G vs. 5G5G (homozygous), 4G5G vs. 5G5G (heterozygous), 4G4G + 4G5G vs. 5G5G (dominant), and 4G4G vs. 5G5G + 5G4G (recessive). Between-study heterogeneity was tested using Q statistics, and *P* < 0.1 was considered statistically significant. The Mantel-Haenszel method for fixed effects and the Der-Simonian and Laird method for random effects were used to estimate pooled effects [16]. We used fixed-effects methods if the result of the Q test was not significant. Otherwise, we calculated the pooled ORs and 95 % CIs assuming a random-effects model. Fixed effects assume that genetic factors have similar effects on disease susceptibility across all studies and that the observed variations between studies are caused by chance alone [17]. The random effects model assumes that different studies may have substantial diversity and assesses both within- and between- study variations [18]. A recently developed measure, I^2^, was used to quantify the inconsistency among the studies’ results with values of 50 % or higher and the large heterogeneity for values of 75 % or higher [19]. The data are shown as the ORs with 95 %CIs, with two-tailed *P*-values; statistical significance was set at *P* < 0.05 (two-tailed). Meta-regression analysis was applied to evaluate the heterogeneity of the studies. Publication bias was conducted statistically via Begg’s and Egger’s bias test, which measures the degree of funnel plot asymmetry [20, 21]. The Begg’s adjusted rank correlation test was used to assess the correlation between test accuracy estimates and their variances. The Egger’s bias test detects funnel plot asymmetry by determining whether the intercept deviates significantly from zero in a regression of the standardized effect estimates against their precision.

## Results

### Search results and characteristics of included studies

**Fig. 1:**
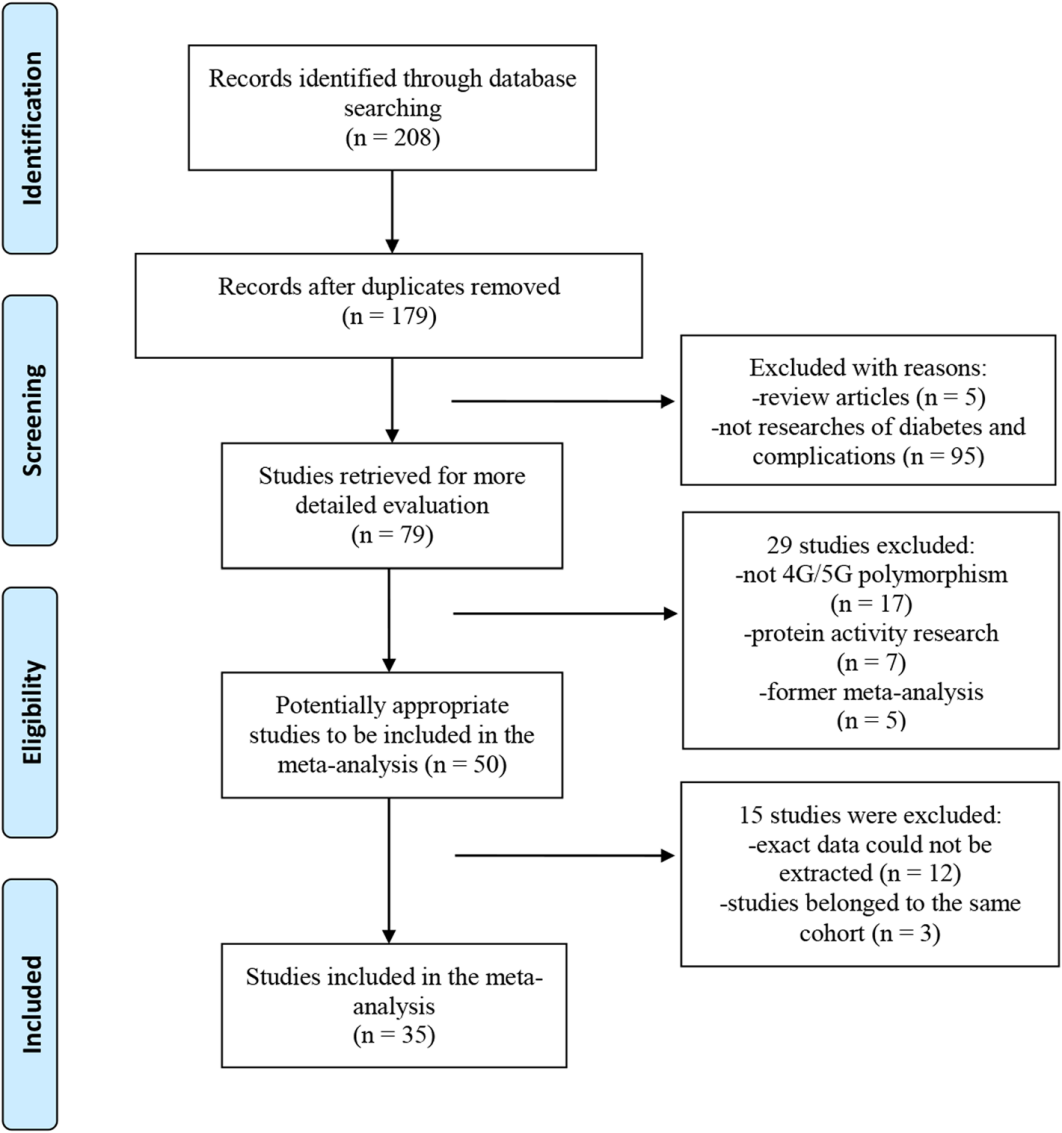
Flow of studies for meta-analysis

The study flow chart is summarized in Fig. 1, the primary literature search identified 208 potentially relevant articles. After exclusion of duplicate or irrelevant articles by reading titles and abstracts, and screening through study results, 50 articles were retrieved for further investigation. Another 15 articles were excluded subsequently after full text evaluation. Finally, a total of 35 studies with 51 comparisons containing 15,341 subjects that met our inclusion and exclusion criteria were included [12, 13, 22–54]. The quality of observational studies is presented in Supplementary Material. All of the studies included in the meta-analysis had high quality in their data outcome and clinical design. Characteristics of included studies were summarized in Table 1.

### Association of SERPINE1 rs1799889 SNP with overall diabetes risk

**Fig. 2:**
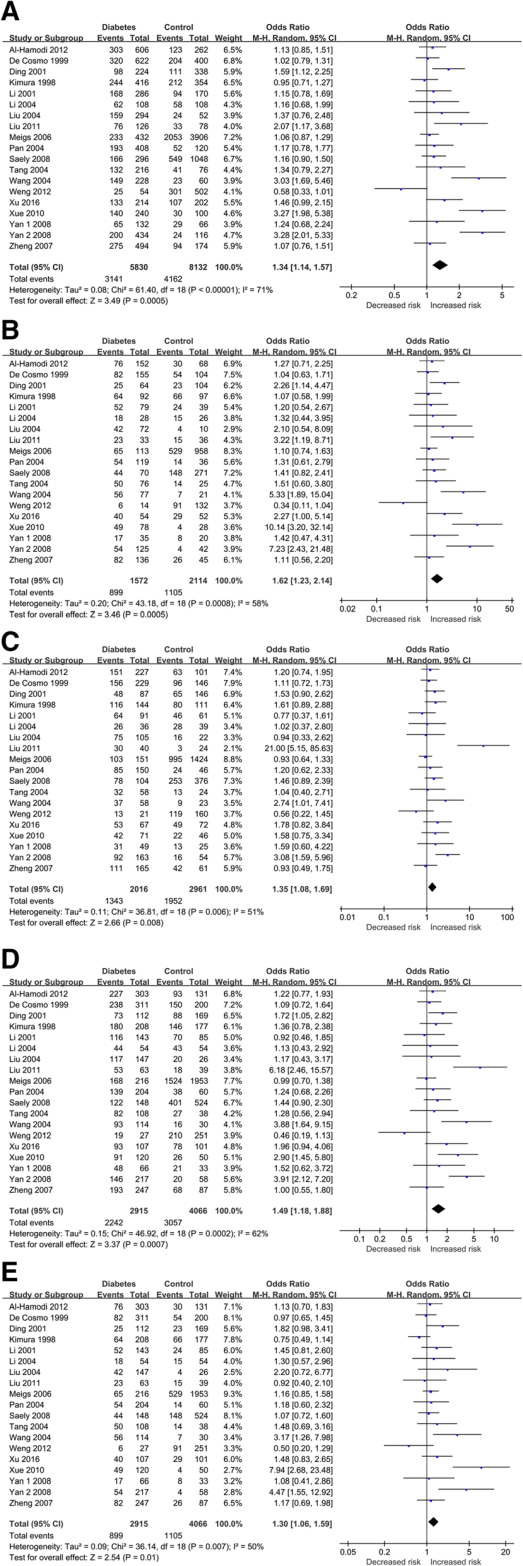
Forest plots of the association between SERPINE1 rs1799889 SNP and diabetes risk. (**A**) allelic model, (**B**) homozygote model, (**C**) heterozygote model, (**D**) dominant model, and (**E**) recessive model

In overall population, our meta-analysis revealed a significant association between the SERPINE1 rs1799889 polymorphism and overall diabetes risk, in allelic (4G vs. 5G: OR = 1.34, 95 % CI = 1.14–1.57, p = 0.00), homozygous (4G4G vs. 5G5G: OR = 1.66, 95 % CI = 1.23–2.14, p = 0.00), heterozygous (4G5G vs. 5G5G: OR = 1.35, 95 % CI = 1.08–1.69, p = 0.00), dominant (4G4G + 4G5G vs. 5G5G: OR = 1.49, 95 % CI = 1.18–1.88, p = 0.00),and recessive (4G4G vs. 5G5G + 5G4G: OR = 1.30, 95 % CI = 1.06–1.59, p = 0.01) models. When analyses were subdivided by ethnicity, no obvious associations were noted for the European using any of the five genetic models. For the Asian subgroup, significant associations were observed in all of the five genetic models (allelic: OR = 1.45, 95 % CI = 1.16–1.82, p = 0.00; homozygous: OR = 1.88, 95 % CI = 1.29–2.75, p = 0.00; heterozygous: OR = 1.47, 95 % CI = 1.08-2.00, p = 0.01; dominant: OR = 1.64, 95 % CI = 1.21–2.24, p = 0.00; recessive: OR = 1.46, 95 % CI = 1.09–1.96, p = 0.01). Results of pooled analyses are summarized and presented in Table 2; Fig. 2.

**Table 2:** Overall and subgroup meta-analysis of the association between SERPINE1 rs1799889 SNP and risk of diabetes

Categories	n	4G vs. 5G			4G4G vs. 5G5G			4G5G vs. 5G5G			4G4G + 4G5G vs. 5G5G			4G4G vs. 5G5G + 5G4G		
		**OR (95% CI)**	***P***	**I**^**2**^**(%)/*P*h**	**OR (95% CI)**	***P*0.00**	**I**^**2**^**(%)/*P*h0.00/0.00**	**OR(95% CI)**	***P***	**I**^**2**^**(%)/*P*h**	**OR(95% CI)**	***P***	**I**^**2**^**(%)/*P*h**	**OR(95% CI)**	***P***	**I**^**2**^**(%)/*P*h**
Overall	19	**1.34 (REM)(1.14–1.57)**	**0.001**	71 %/0.001	**1.62 (REM)(1.23–2.14)**	**0.001**	58 %/0.001	**1.35 (REM)(1.08–1.69)**	**0.001**	51 %/0.01	**1.49 (REM)(1.18–1.88)**	**0.001**	62 %/0.001	**1.30 (REM)(1.06–1.59)**	**0.01**	50 %/0.01
**Subgroup (by population)**																
European	3	1.07(0.94–1.23)	0.31	0 %/0.76	1.15(0.88–1.50)	0.31	0 %/0.69	1.10(0.86–1.40)	0.45	7 %/0.34	1.12(0.89–1.40)	0.35	0 %/0.43	1.08(0.88–1.33)	0.46	0 %/0.78
Asian	15	**1.45 (REM)(1.16–1.82)**	**0.001**	74 %/0.001	**1.88 (REM)(1.29–2.75)**	**0.001**	62 %/0.01	**1.47 (REM)(1.08-2.00)**	**0.01**	56 %/0.01	**1.64 (REM)(1.21–2.24)**	**0.001**	63 %/0.001	**1.46(1.09–1.96)**	**0.01**	58 %/0.001
Others	1	1.13(0.85–1.51)	0.41	N/A	1.27(0.71–2.25)	0.42	N/A	1.20(0.74–1.95)	0.47	N/A	1.22(0.77–1.93)	0.39	N/A	1.13(0.70–1.83)	0.63	N/A

n: study numbers, OR: odds ratio, CI: confidence interval, bold values represent statistically significant findings, *P*h: *P* heterogeneity (*P* < 0.1 was considered as a significant difference), REM: Random Effects Model

### Association of SERPINE1 rs1799889 SNP with DR risk

**Fig. 3:**
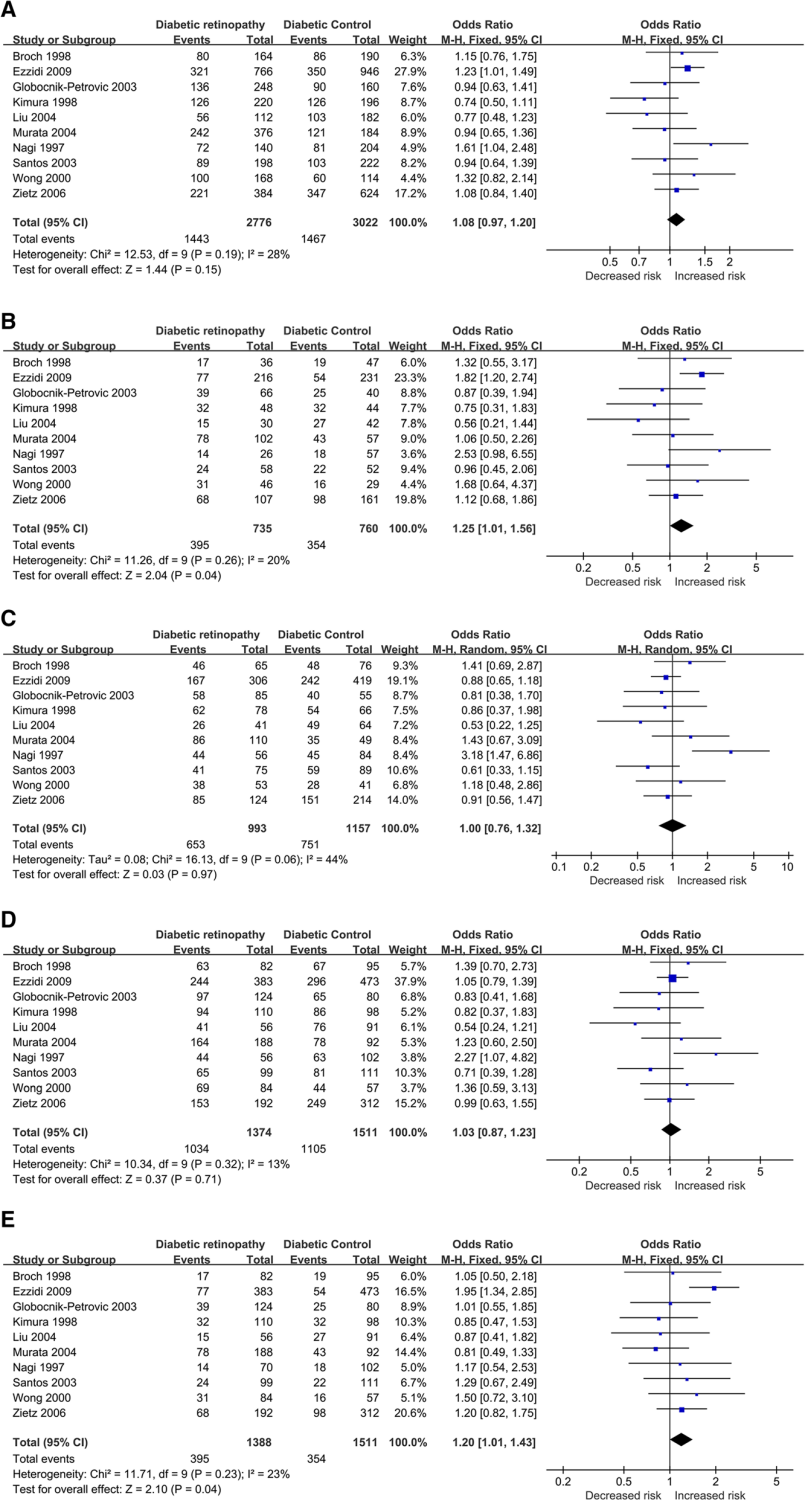
Forest plots of the association between SERPINE1 rs1799889 SNP and DR risk. (**A**) allelic model, (**B**) homozygote model, (**C**) heterozygote model, (**D**) dominant model, and (**E**) recessive model (DR: diabetic retinopathy)

In overall population, a significant association between the SERPINE1 rs1799889 polymorphism and DR risk was observed in homozygous (4G4G vs. 5G5G: OR = 1.25, 95 % CI = 1.01–1.56, p = 0.04) and recessive (4G4G vs. 5G5G + 5G4G: OR = 1.20, 95 % CI = 1.01–1.43, p = 0.04) models, but no association was found in the other three genetic models. For the European subgroup, a significant association was revealed by homozygous (OR = 1.32, 95 % CI = 1.02–1.72, p = 0.04) and recessive model (OR = 1.38, 95 % CI = 1.11–1.71, *p* < 0.01), but no association was observed in the allelic, heterozygote, and dominant models. No significant associations were indicated among Asian descent in all genetic models. Results of pooled analyses are summarized and presented in Table 3; Fig. 3.

**Table 3:** Overall and subgroup meta-analysis of the association between SERPINE1 rs1799889 SNP and risk of diabetic retinopathy

Categories	n	4G vs. 5G			4G4G vs. 5G5G			4G5G vs. 5G5G			4G4G + 4G5G vs. 5G5G			4G4G vs. 5G5G + 5G4G		
		OR (95% CI)	*P*	I^2^ (%)/ *P*h	OR (95% CI)	*P*0.00	I^2^ (%)/ *P*h0.00/0.00	OR(95% CI)	*P*	I^2^ (%)/ *P*h	OR(95% CI)	*P*	I^2^ (%)/ *P*h	OR(95% CI)	*P*	I^2^ (%)/ *P*h
Overall	10	1.08(0.97–1.20)	0.15	28 %/0.19	**1.25(1.01–1.56)**	**0.04**	23 %/0.23	1.00 (REM)(0.76–1.32)	0.97	44 %/0.06	1.03(0.87–1.23)	0.71	13 %/0.32	**1.20(1.01–1.43)**	**0.04**	23 %/0.23
Subgroup (by population)																
European	5	1.12(0.98–1.27)	0.09	0 %/0.66	**1.32(1.02–1.72)**	**0.04**	26 %/0.25	0.88(0.71–1.09)	0.24	0 %/0.55	1.00(0.82–1.22)	0.98	0 %/0.63	**1.38(1.11–1.71)**	**0.001**	26 %/0.25
Asian	4	0.90(0.73–1.11)	0.34	22 %/0.28	0.94(0.60–1.45)	0.77	0 %/0.56	0.95(0.63–1.45)	0.83	5 %/0.37	0.94(0.63–1.39)	0.75	6 %/0.36	0.93(0.68–1.26)	0.63	0 %/0.56
Others	1	1.61(1.04–2.48)	0.03	N/A	2.53(0.98–6.55)	0.06	N/A	3.18(1.47–6.86)	0.003	N/A	2.27(1.07–4.82)	0.03	N/A	1.17(0.54–2.53)	0.70	N/A

n: study numbers, OR: odds ratio, CI: confidence interval, bold values represent statistically significant findings, *P*h: *P* heterogeneity (*P* < 0.1 was considered as a significant difference), REM: Random Effects Model

### Association of SERPINE1 rs1799889 SNP with diabetic CVD risk

**Fig. 4:**
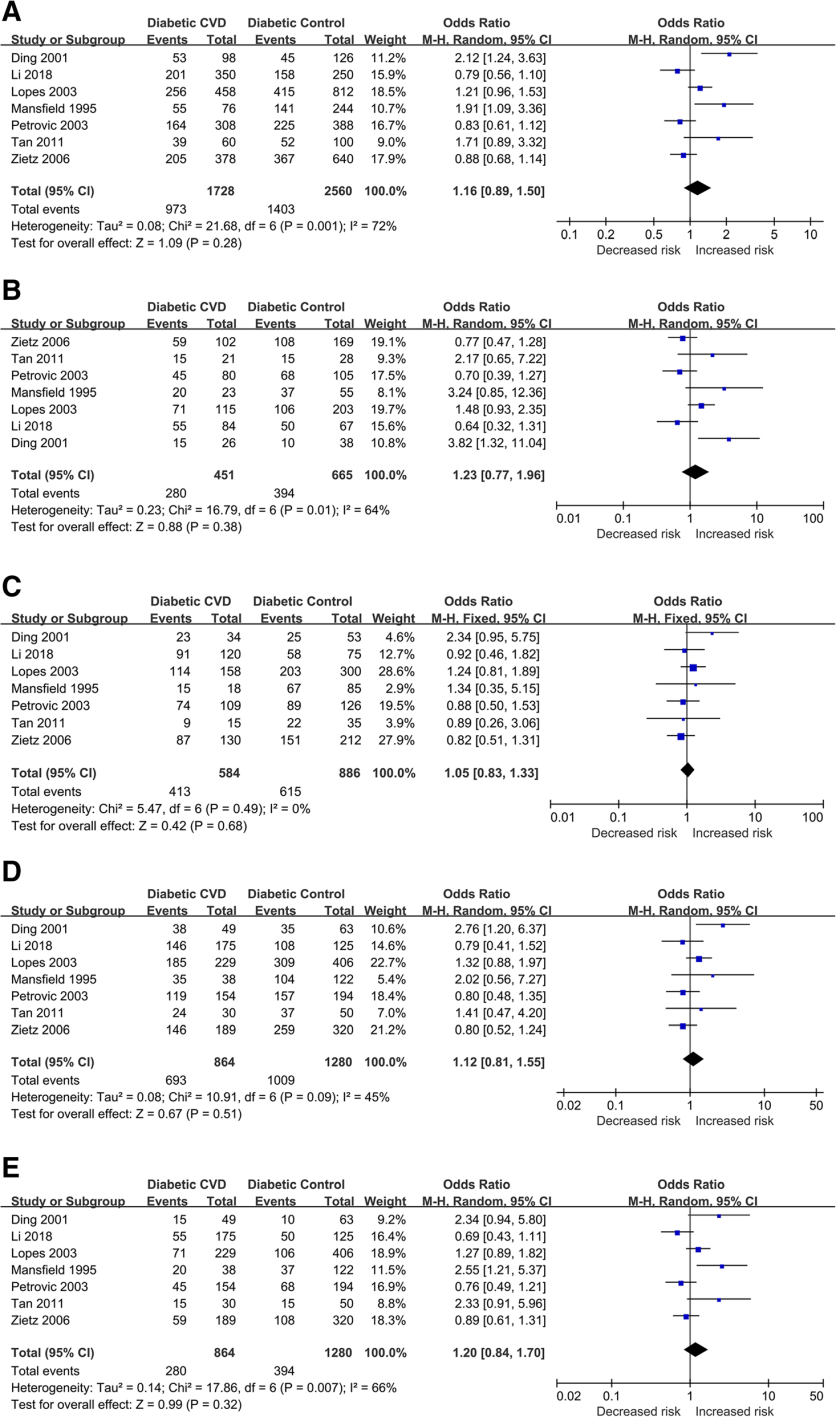
Forest plots of the association between SERPINE1 rs1799889 SNP and diabetic CVD risk. (**A**) allelic model, (**B**) homozygote model, (**C**) heterozygote model, (**D**) dominant model, and (**E**) recessive model (CVD: cardiovascular disease)

No significant association was implied between the SERPINE1 rs1799889 polymorphism and overall diabetic CVD risk in any genetic models. Additionally, after ethnicity stratification, no significant association was revealed either in European or Asian descent. Results of pooled analyses are summarized and presented in Table 4; Fig. 4.

**Table 4:** Overall and subgroup meta-analysis of the association between SERPINE1 rs1799889 SNP and risk of diabetic CVD

Categories	n	4G vs. 5G			4G4G vs. 5G5G			4G5G vs. 5G5G			4G4G + 4G5G vs. 5G5G			4G4G vs. 4G5G + 5G5G		
		OR (95% CI)	*P*	I^2^ (%)/ *P*h	OR (95% CI)	*P*0.00	I^2^ (%)/ *P*h0.00/0.00	OR(95% CI)	*P*	I^2^ (%)/ *P*h	OR(95% CI)	*P*	I^2^ (%)/ *P*h	OR(95% CI)	*P*	I^2^ (%)/ *P*h
Overall	7	1.16(0.89–1.50)	0.28	72 %/0.001	1.23(0.77–1.96)	0.38	64 %/0.01	1.05 (FEM)(0.83–1.33)	0.68	0 %/0.49	1.12(0.81–1.55)	0.51	45 %/0.09	1.20(0.84–1.70)	0.32	66 %/0.01
Subgroup (by population)																
European	4	1.07(0.81–1.42)	0.63	70 %/0.02	1.08(0.65–1.80)	0.77	62 %/0.05	1.00 (FEM)(0.77–1.31)	0.97	0 %/0.56	1.12 (FEM)(0.89–1.40)	0.35	0 %/0.43	1.13(0.76–1.68)	0.54	67 %/0.03
Asian	3	1.37(0.69–2.73)	0.37	82 %/0.001	1.64(0.52–5.23)	0.40	76 %/0.02	1.24(0.66–2.33)	0.50	32 %/0.23	1.41(0.63–3.13)	0.40	63 %/0.07	1.45(0.57–3.65)	0.43	77 %/0.01

n: study numbers, OR: odds ratio, CI: confidence interval, bold values represent statistically significant findings, *P*h: *P* heterogeneity (*P* < 0.1 was considered as a significant difference), FEM: Fix Effects Model, CVD: Cardiovascular disease

### Association of SERPINE1 rs1799889 SNP with DN risk

**Fig. 5:**
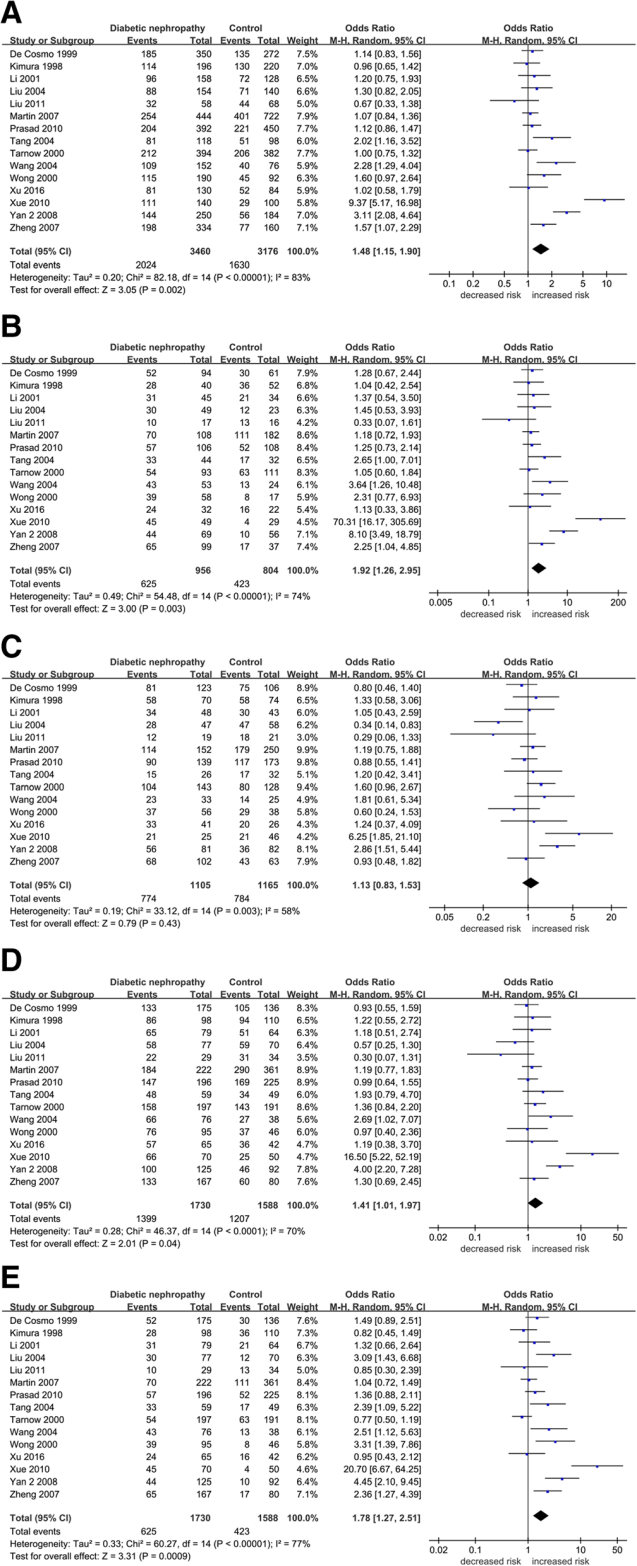
Forest plots of the association between SERPINE1 rs1799889 SNP and DN risk. (**A**) allelic model, (**B**) homozygote model, (**C**) heterozygote model, (**D**) dominant model, and (**E**) recessive model (DN: diabetic nephropathy)

In overall population, significant associations were shown between the SERPINE1 rs1799889 polymorphism and overall diabetic nephropathy risk, in allelic (4G vs. 5G: OR = 1.48, 95 % CI = 1.15–1.90, p = 0.00), homozygous (4G4G vs. 5G5G: OR = 1.92, 95 % CI = 1.26–2.95, p = 0.00), dominant (4G4G + 4G5G vs. 5G5G: OR = 1.41, 95 % CI = 1.01–1.97, p = 0.04), and recessive (4G4G vs. 5G5G + 5G4G: OR = 1.78, 95 % CI = 1.27–2.51, p = 0.00) models. After subdivided by ethnicity, remarkable associations were observed in allelic (OR = 1.70, 95 % CI = 1.17–2.47, p = 0.01), homozygous (OR = 2.46, 95 % CI = 1.30–4.66, p = 0.01), and recessive (OR = 2.24, 95 % CI = 1.40–3.59, p = 0.00) models for Asian subgroup. On the contrary, no obvious associations were noted for the European using any of the five genetic models. Results of pooled analyses are summarized and presented in Table 5; Fig. 5.

**Table 5:** Overall and subgroup meta-analysis of the association between SERPINE1 rs1799889 SNP and risk of diabetic nephropathy

Categories	n	4G vs. 5G			4G4G vs. 5G5G			4G5G vs. 5G5G			4G4G + 4G5G vs. 5G5G			4G4G vs. 5G5G + 5G4G		
		OR (95 %CI)	*P*	I^2^ (%)/ *P*h	OR (95 %CI)	*P*0.00	I^2^ (%)/ *P*h0.00/0.00	OR(95 %CI)	*P*	I^2^ (%)/ *P*h	OR(95 %CI)	*P*	I^2^ (%)/ *P*h	OR(95 %CI)	*P*	I^2^ (%)/ *P*h
Overall	15	**1.48 (REM)(1.15–1.90)**	**0.001**	83 %/0.001	**1.92 (REM)(1.26–2.95)**	**0.001**	74 %/0.001	1.13 (REM)(0.83–1.53)	0.43	58 %/0.001	**1.41 (REM)(1.01–1.97)**	**0.04**	70 %/0.001	**1.78 (REM)(1.27–2.51)**	**0.001**	77 %/0.001
Subgroup (by population)																
European	3	1.06(0.91–1.24)	0.45	0 %/0.82	1.16(0.84–1.60)	0.37	0 %/0.90	1.17(0.88–1.57)	0.28	38 %/0.20	1.16(0.88–1.53)	0.28	0 %/0.59	1.04(0.74–1.46)	0.84	46 %/0.15
Asian	11	**1.70 (REM)(1.17–2.47)**	**0.01**	84 %/0.001	**2.46 (REM)(1.30–4.66)**	**0.01**	76 %/0.001	1.15 (REM)(0.71–1.86)	0.56	65 %/0.001	1.59 (REM)(0.94–2.69)	0.08	75 %/0.001	**2.24 (REM)(1.40–3.59)**	**0.001**	75 %/0.001
Others	1	1.12(0.86–1.47)	0.40	N/A	1.25(0.73–2.14)	0.41	N/A	0.88(0.55–1.41)	0.59	N/A	0.99(0.64–1.55)	0.98	N/A	1.36(0.88–2.11)	0.16	N/A

n: study numbers, OR: odds ratio, CI: confidence interval, bold values represent statistically significant findings, *P*h: *P* heterogeneity (*P* < 0.1 was considered as a significant difference), REM: Random Effects Model

### Meta-regression analysis

A meta-regression analysis for the discovery of potential origins of heterogeneity, such as study type, published years, sample sizes, age, gender, ethnicity and outcomes, was conducted. Single covariates were added in the allelic, homozygous, heterozygous, dominant and recessive models. However, the results of meta-regression indicated that none of the above sources contributed to the heterogeneity across all studies of the association between SERPINE1 rs1799889 polymorphism and diabetic vascular susceptibility, since all the *p* values calculated were larger than 0.05.

### Sensitivity analysis and publication bias

Sensitivity analysis with stratified analyses were conducted to examine the stability of our meta-analysis results. The high heterogeneity in some of the genetic models was obvious among studies except for the association with DR risk. On the association with DM and DN risk, a heterogeneity was detected within the overall analysis for the allelic model. On the association with DM, DR and DN risks, the heterogeneity in any genetic model was not significantly varied after either sensitivity analysis or sub-group analysis by ethnicity stratification. On the association with diabetic CVD risk, heterogeneity was noted for allelic/homozygote/recessive models, except for the European sub-group. After the sensitivity analysis, the study from Li et al. [12] were mainly responsible for the observed heterogeneity.

Potential publication bias in the current study was evaluated with Begg’s and Egger’s bias test. Publication bias was noted within DM sub-group with Egger test and DN sub-group for recessive model. Except for that, no obvious publication bias was observed in other comparisons, which confirmed that the results our meta-analysis presented were statistically robust (Table 6).

**Table 6:** Publication bias assessment of this meta-analysis

Genetic model	Egger’s test		Begg’s test	
	t-value	*p*	t-value	*p*
**Diabetes**				
Allelic model	2.96	*0.01*	2.72	*0.01*
Homozygote model	2.99	*0.01*	2.96	*0.001*
Heterozygote model	3.11	*0.01*	2.11	0.04
Dominant model	2.48	*0.02*	1.99	0.05
Recessive model	2.23	*0.03*	1.87	0.06
**Diabetic retinopathy**				
Allelic model	-0.98	0.36	0.00	1.00
Homozygote model	-1.88	0.10	0.36	0.72
Heterozygote model	0.74	0.48	0.54	0.59
Dominant model	0.04	0.97	0.00	1.00
Recessive model	-1.39	0.20	0.00	1.00
**Diabetic CVD**				
Allelic model	1.88	0.12	1.20	0.23
Homozygote model	1.49	0.20	0.60	0.55
Heterozygote model	0.62	0.56	0.90	0.37
Dominant model	1.13	0.31	0.90	0.37
Recessive model	1.88	0.12	0.30	0.76
**Diabetic nephropathy**				
Allelic model	1.18	0.09	1.98	0.05
Homozygote model	1.63	0.13	1.48	0.14
Heterozygote model Dominant model	-0.11 0.61	0.91 0.55	0.00 0.69	1.00 0.49
Recessive model	3.05	*0.01*	2.18	*0.03*

*P* ≺ 0.05 was considered as a significant difference

## Discussion

The current meta-analysis suggests that the SERPINE1 rs1799889 4G polymorphism possesses a genetic modulatory function in overall DM populations and in diabetic renal vascular complications, which can be ethnically divergent according to the results. Genetic factors have long been considered a substantial determinant within the diabetic physical-sociocultural environment [55]. Positive family history might attribute a 2- to 4-fold increase in risk for diabetes [56]. The DCCT (Diabetes Control and Complications Trial) [57] and the EDIC (Epidemiology of Diabetes Interventions and Complications) [58] established that hyperglycemia is modified by both genetic determinants of individual susceptibility and by independent accelerating factors. Recently, large-scale genome wide association studies (GWAS) [59, 60] have identified hundreds of genetic risk variants, which in aggregate could explain the substantial role of genetic predisposition in DM. Additionally, one recent exome sequencing study [61] discovered additional genes and pathways for future target gene prioritization efforts and complications in DM [60]. Overall, the evidence jointly supports the theory that genetic factors significantly account for the pathogenesis of DM and its complications.

PAI-1 is a serine protease inhibitor protein encoded by the SERPINE1 gene that plays an important role in regulating fibrinolysis and thrombosis by inhibiting the activity of tissue plasminogen activator and urokinase plasminogen activator, whose activation is driven by tissue-type plasminogen activator (tPA) cleavage of plasminogen [62]. Previous human and animal PAI-1 studies have confirmed its effect on hemostasis and thrombolysis, where suppressing PAI-1 activity would resulted in a reduction of thrombus formation while activation of the PAI-1 promoted thrombus formation [63]. Classic studies have confirmed that high plasma levels of PAI-1 are associated with an increased risk of cardiovascular diseases [64, 65], and SERPINE1 allelic variations are also associated with the pathogenesis of metabolic syndrome, insulin resistance, and diabetes [66–68]. To date, several SERPINE1 polymorphisms have been identified, of which the SERPINE1 rs1799889–4G/5G insertion-deletion variant has been most consistently implicated with the plasma level of PAI-1 [68]. Unlike the 5G allele, which binds a transcription repressor protein resulting in low PAI-1 expression, the 4G allele does not bind a transcription repressor, thus conferring a “high PAI-1 expressor” nature to the allele [9]. In diabetic populations, PAI-1 levels are particularly connected to elevated fasting insulin levels and triglycerides, and inhibition of PAI-1 may have merit in patients at high cardiovascular risk [69].

Previous studies of the distribution of the SERPINE1 rs1799889 SNP have been controversial concerning the susceptibility of diabetes among various populations. Saely et al. [37] demonstrated no significant difference in the SERPINE1 4G/5G polymorphism between nondiabetic control subjects and diabetic patients. In contrast, Al-Hamodi et al. [41] suggested that the dominant and additive models showed a weak association with T2DM. Nagi et al. [23] reported preliminary findings indicating that in Pima Indians with type 2 diabetes, the presence of the 4G allele was associated with a higher risk of diabetic retinopathy. However, Santos et al. [34] indicated that the 4G/5G polymorphism was not related to the presence of DR in Euro-Brazilian patients. While Ezzidi et al. [40] identified that genetic variations served as risk factors for DR but not DR severity. Tarnow et al. [46] suggested that the SERPINE1 4G/5G polymorphism might not contribute to the genetic susceptibility to diabetic nephropathy or retinopathy. In contrast, Prasad et al. [48] and Xu et al. [13] demonstrated major associations with the SERPINE1 rs1799889 4G polymorphism and the progression of diabetic nephropathy. Mansfield et al. [22] and Lopes et al. [31] have proved the synergistic effect between the SERPINE1 4G/5G polymorphism and CVD, suggesting its potential correlation with insulin-resistance and obesity. Nevertheless, Petrovic et al. [29] found no association between this polymorphism and myocardial infarction.

Our results revealed an obvious difference in the association of the SERPINE1 rs1799889 SNP among individuals with Asian and European descent, implying that the heterogeneity is based on ethnicity. Concerning the association with diabetes risk, our results suggested that the 4G polymorphism is a genetic risk factor in overall populations. Moreover, after stratification by ethnicity, the results revealed a remarkable association with Asian descent, while no association was found for European diabetic populations. A previous meta-analysis showed different results [11]. Regarding the association with DR risk, our results differed from Zhang et al. [10] but were in concordance with Xu et al. [11]. In our analysis, we included a novel German study [36]. Additionally, both random and fixed effects model was adapted to demonstrate less bias and to confirm a robust conclusion. Since our meta-analysis has included recent published studies and larger sample sizes, we suppose it could provide better reliability. We hypothesize that these factors might contribute to the disparities with other studies. Concerning the association with diabetic CVD risk, our results coincided with a previous analysis [11], which proved no significant association despite the inclusion of recent studies [12]. This result was to some extent disparate from other analyses concerning PAI-1 polymorphisms in atherosclerotic diseases [70] and suggests that the underlying mechanism for the SERPINE1 4G/5G polymorphism might be conducted through different pathways in diabetic CVD. Concerning the association with DN risk, our results indicated a strong linkage between SERPINE1 4G polymorphism and DN risk in the overall and Asian populations. This is consistent with former studies [25, 71] and further implies that heterogeneity is affected by ethnicity. Moreover, insufficient genetic data in mix ethnicities could limit the possibility of further discussion regarding this population, which to a considerable extent could alter the overall analyses. To our knowledge, the current meta-analysis includes the largest sample size to date with the most extensive case-control studies, and demonstrates an ethnicity-based evaluation for different results among studies. The association with ischemic stroke in the diabetic population was not further evaluated in the present study owing to limitations of available trails, but would be an important topic for consideration in future studies concerning diabetic atherothrombotic complications. In addition, future investigations are also warranted to discover the possible functions of other SERPINE1 gene polymorphisms in DM and its complications.

Since our meta-analysis was conducted with stratified ethnicity, the origins of heterogeneity must be given thorough discussion. In our analysis, heterogeneity was revealed among people of Asian descent both in the CVD and DN subgroups. We speculate that the sources of heterogeneity in studies might include age and gender proportion, ethnic traits, environmental factors, medication status, health care quality and cultural differences. A meta-regression analysis was done by study type, published years, age, gender, ethnicity, sample sizes, and outcomes. However, the results did not indicate the sources of heterogeneity, since all the p values calculated above were larger than 0.05. As we speculated, meta-regression is usually conducted in studies with larger sample sizes and study sub-groups, whose effect might be restrained in this case. Moreover, the gene-gene and gene-environmental interactions might also trigger the heterogeneity of genetic effects between individual studies.

There were several limitations included in our meta-analysis: (1) insufficient genotyping data of SERPINE1 rs1799889 SNP in mix ethnicity, which limited the possibility to further discussions regarding this population, and (2) potential heterogeneity of study variables, such as the biological parameters of study subjects, clinical history, medication compliance, other diabetic complications, etc. and (3) the Begg’s and Egger’s test have given some potential publication bias, indicating the importance of a well-matched case-control study population. (4) Sample size is another limitation, some of the original studies analyzed presented relatively small control groups, and the minor allele frequency (G or 5G; MAF) of the control populations analyzed are heterogeneous, between 34.2 and 71 %, including among studies in the same ethnicity group and also in the same study among different analyzed groups. (5) Insufficiency of original studies of type 1 DM has restrained a further subgroup analysis concerning the classification of DM.

## Conclusions

Collectively, our meta-analysis demonstrates that the SERPINE1 rs1799889 4G polymorphism may outstand for serving as a genetic synergistic factor in overall DM populations, and overall DN populations. Moreover, it can be positively associated with increased DM and DN risks for individuals with Asian descent. The association of SERPINE1 rs1799889 polymorphisms and DR or diabetic CVD risks was not revealed by our meta-analysis. However, future studies with multiple ethnicities and rigorous designs are still in-need to confirm our conclusions.

## Supplementary information

Additional file 1: **Supplementary Table 1.** Newcastle–Ottawa scale (NOS) for assessing quality of observational studies.

Additional file 2: **Supplementary Table 2.** Search strategy for PubMed.

Additional file 3: **Supplementary Fig. 1.** Cumulative meta-analysis of the chronologic integration between SERPINE1 rs1799889 SNP and diabetes risk. (**A**) allelic model, (**B**) homozygote model, (**C**) heterozygote model, (**D**) dominant model, and (**E**) recessive model.

Additional file 4: **Supplementary Fig. 2.** Begg’s funnel plot of bias for studies of the association between SERPINE1 rs1799889 SNP and diabetes risk. (**A**) allelic model, (**B**) homozygote model, (**C**) heterozygote model, (**D**) dominant model, and (**E**) recessive model.

Additional file 5: **Supplementary Fig. 3.** Begg’s funnel plot of bias for studies of the association between SERPINE1 rs1799889 SNP and DR risk. (**A**) allelic model, (**B**) homozygote model, (**C**) heterozygote model, (**D**) dominant model, and (**E**) recessive model.

Additional file 6: **Supplementary Fig. 4.** Begg’s funnel plot of bias for studies of the association between SERPINE1 rs1799889 SNP and CVD risk. (**A**) allelic model, (**B**) homozygote model, (**C**) heterozygote model, (**D**) dominant model, and (**E**) recessive model.

Additional file 7: **Supplementary Fig. 5.** Begg’s funnel plot of bias for studies of the association between SERPINE1 rs1799889 SNP and DN risk. (**A**) allelic model, (**B**) homozygote model, (**C**) heterozygote model, (**D**) dominant model, and (**E**) recessive model.

## Data Availability

The data analysed during the current meta-analysis is included in this published article and its supplementary information files, and other relevant data is available from the corresponding author on reasonable request.

## Abbreviations

DM: diabetes mellitus
SERPINE1: serine protease inhibitor-1
SNP: single nucleotide polymorphism
PAI-1: plasminogen activator inhibitor-1
PRISMA: Preferred Reporting Items for Systematic Reviews and Meta-Analyses
DR: diabetic retinopathy
DN: diabetic nephropathy
CAD/CHD: coronary artery/heart disease
CVD: cardiovascular disease
OR: odds ratio
CI: confidence interval
DCCT: Diabetes Control and Complications Trial
EDIC: Epidemiology of Diabetes Interventions and Complications
GWAS: Genome Wide Association Studies
tPA: tissue-type plasminogen activator
