# Supplementary material for: The susceptibility of SERPINE1 rs1799889 SNP in diabetic vascular complications: a meta-analysis of fifty-one case-control studies
Source: BMC Endocr Disord. 2021 Sep 30;21:195. doi: 10.1186/s12902-021-00837-z (PMC8482645; doi:10.1186/s12902-021-00837-z)
Supplement: Supplementary file 1 — Supplementary Table 1. Newcastle–Ottawa scale (NOS) for assessing quality of observational studies. [file 12902_2021_837_MOESM1_ESM.docx]

Supplementary Table. Newcastle–Ottawa scale (NOS) for assessing quality of observational studies.

| Study | Selection | | | | Comparability of the cohort | Outcome | | | Total score |
| --- | --- | --- | --- | --- | --- | --- | --- | --- | --- |
|  | Representativeness of the exposed cohort | Selection of the nonexposed cohort | Ascertainment of exposure | Outcome not present at baseline |  | Assessment of outcome | Enough follow-up duration | Adequate follow-up |  |
| Mansfield et al | * | * | * | * | ** | * | - | - | 7 |
| Nagi et al | * | * | * | * | ** | * | * | * | 9 |
| Broch et al | * | * | * | * | ** | * | - | - | 7 |
| Kimura et al | * | * | * | * | ** | * | * | - | 8 |
| De Cosmo et al  Tarnow et al | *  * | *  * | *  * | *  * | **  ** | *  * | *  * | -  - | 8  8 |
| Wong et al | * | * | * | * | ** | * | - | * | 8 |
| Ding et al  Li et al | *  * | *  * | *  * | *  * | **  ** | *  * | -  * | -  * | 7  9 |
| Petrovic et al | * | * | * | * | ** | * | - | - | 7 |
| Santos et al  Globocnik-P et al  Lopes et al  Liu et al  Pan et al  Li et al  Murata et al  Wang et al  Tang et al  Meigs et al  Zietz et al  Martin et al  Zheng et al  Saely et al  Yan et al 1  Yan et al 2  Ezzidi et al  Xue et al  Prasad et al  Tan et al  Liu et al  Al-Hamodi et al  Weng et al  Xu et al  Li et al | *  *  *  *  *  *  *  *  *  *  *  *  *  *  *  *  *  *  *  *  *  *  *  *  * | *  *  *  *  *  *  *  *  *  *  *  *  *  *  *  *  *  *  *  *  *  *  *  *  * | *  *  *  *  *  *  *  *  *  *  *  *  *  *  *  *  *  *  *  *  *  *  *  *  * | *  *  *  *  *  *  *  *  *  *  *  *  *  *  *  *  *  *  *  *  *  *  *  *  * | **  **  **  **  **  **  **  **  **  **  **  **  **  **  **  **  **  **  **  **  **  **  **  **  ** | *  *  *  *  *  *  *  *  *  *  *  *  *  *  *  *  *  *  *  *  *  *  *  *  * | -  -  -  *  *  -  -  *  *  *  *  *  *  *  -  *  -  *  -  *  *  -  *  *  * | -  -  -  -  -  -  -  -  -  *  -  -  *  *  -  -  -  *  -  -  *  -  *  -  - | 7  7  7  8  8  7  7  8  8  9  8  8  9  9  7  8  7  9  7  8  9  7  9  8  8 |

The scale assigns 4 points for selection, 2 points for comparability and 3 points for outcome. Score of 5 to 6 considered as moderate quality and 7 to 9 as high quality.
