# Supplementary material for: The susceptibility of SERPINE1 rs1799889 SNP in diabetic vascular complications: a meta-analysis of fifty-one case-control studies
Source: BMC Endocr Disord. 2021 Sep 30;21:195. doi: 10.1186/s12902-021-00837-z (PMC8482645; doi:10.1186/s12902-021-00837-z)
Supplement: Supplementary file 2 — Supplementary Table 2. Search strategy for PubMed. [file 12902_2021_837_MOESM2_ESM.docx]

**Search Strategy for PubMed**

**#1 Search diabetes mellitus [Title/Abstract]**

**#2 Search DM [Title/Abstract]**

**#3 Search diabetes [Title/Abstract]**

**#4 Search diabetic [Title/Abstract]**

**#5 Search #1 OR #2 OR #3 OR #4**

**#6 Search plasminogen activator inhibitor-1 [Title/Abstract]**

**#7 Search PAI-1 [Title/Abstract]**

**#8 Search SERPINE1 [Title/Abstract]**

**#9 Search #6 OR #7 OR #8**

**#10 Search polymorphism [Title/Abstract]**

**#11 Search variants [Title/Abstract]**

**#12 Search variations [Title/Abstract]**

**#13 Search genotype [Title/Abstract]**

**#14 Search #10 OR #11 OR #12 OR #13**

**#15 Search 4G [Title/Abstract]**

**#16 Search 5G [Title/Abstract]**

**#17 Search 4G/5G [Title/Abstract]**

**#18 Search #15 OR #16 OR #17**

**#19 Search #5 AND #9 AND #14 AND #18**

**#20 Search coronary heart disease [Title/Abstract]**

**#21 Search coronary artery disease [Title/Abstract]**

**#22 Search cardiovascular disease [MeSH Terms]**

**#23 Search #20 OR #21 OR #22**

**#24 Search #19 AND #23**

**#25 Search nephropathy [Title/Abstract]**

**#26 Search renal disease [Title/Abstract]**

**#27 Search #25 OR #26**

**#28 Search #19 AND #28**

**#29 Search retinopathy [Title/Abstract]**

**#30 Search diabetic retinopathy [MeSH Terms]**

**#31 Search #29 OR #30**

**#32 Search #19 AND #31**
