# Supplementary figures and images for: The susceptibility of SERPINE1 rs1799889 SNP in diabetic vascular complications: a meta-analysis of fifty-one case-control studies
Source: BMC Endocr Disord. 2021 Sep 30;21:195. doi: 10.1186/s12902-021-00837-z (PMC8482645; doi:10.1186/s12902-021-00837-z)

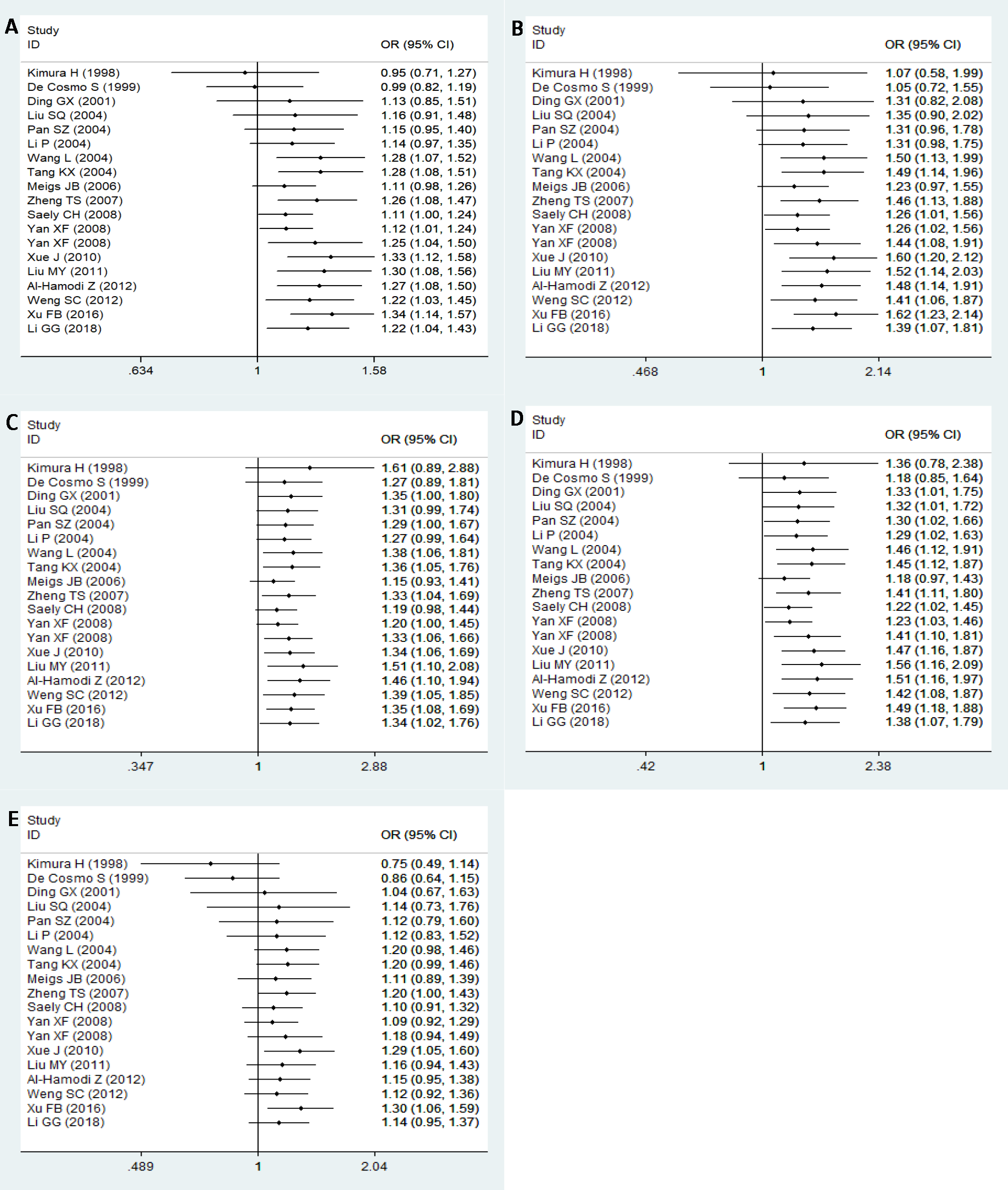

Supplement: Supplementary file 3 — Supplementary Fig. 1. Cumulative meta-analysis of the chronologic integration between SERPINE1 rs1799889 SNP and diabetes risk. (A) allelic model, (B) homozygote model, (C) heterozygote model, (D) dominant model, and (E) recessive model. [file 12902_2021_837_MOESM3_ESM.tif]

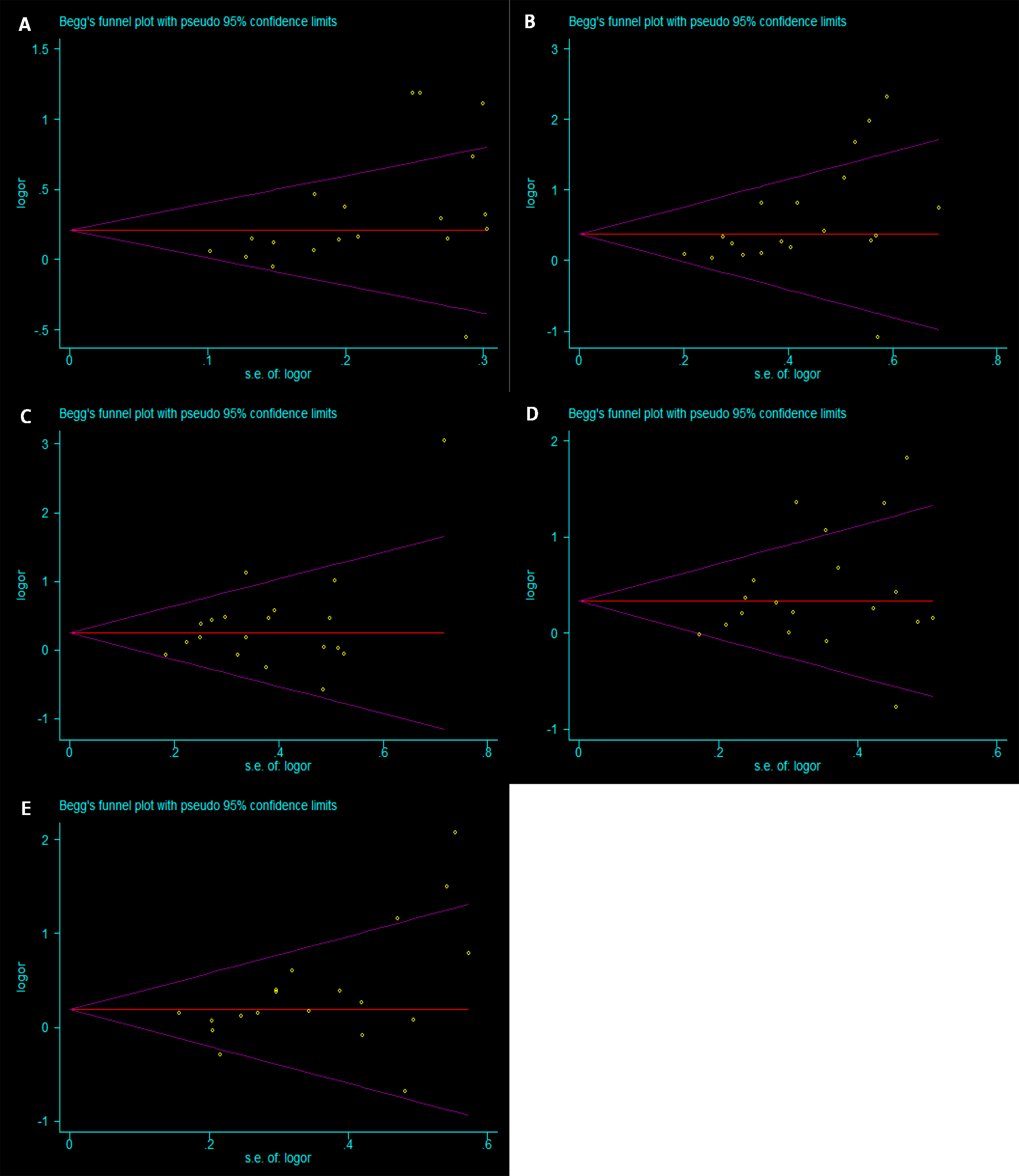

Supplement: Supplementary file 4 — Supplementary Fig. 2. Begg’s funnel plot of bias for studies of the association between SERPINE1 rs1799889 SNP and diabetes risk. (A) allelic model, (B) homozygote model, (C) heterozygote model, (D) dominant model, and (E) recessive model. [file 12902_2021_837_MOESM4_ESM.tif]

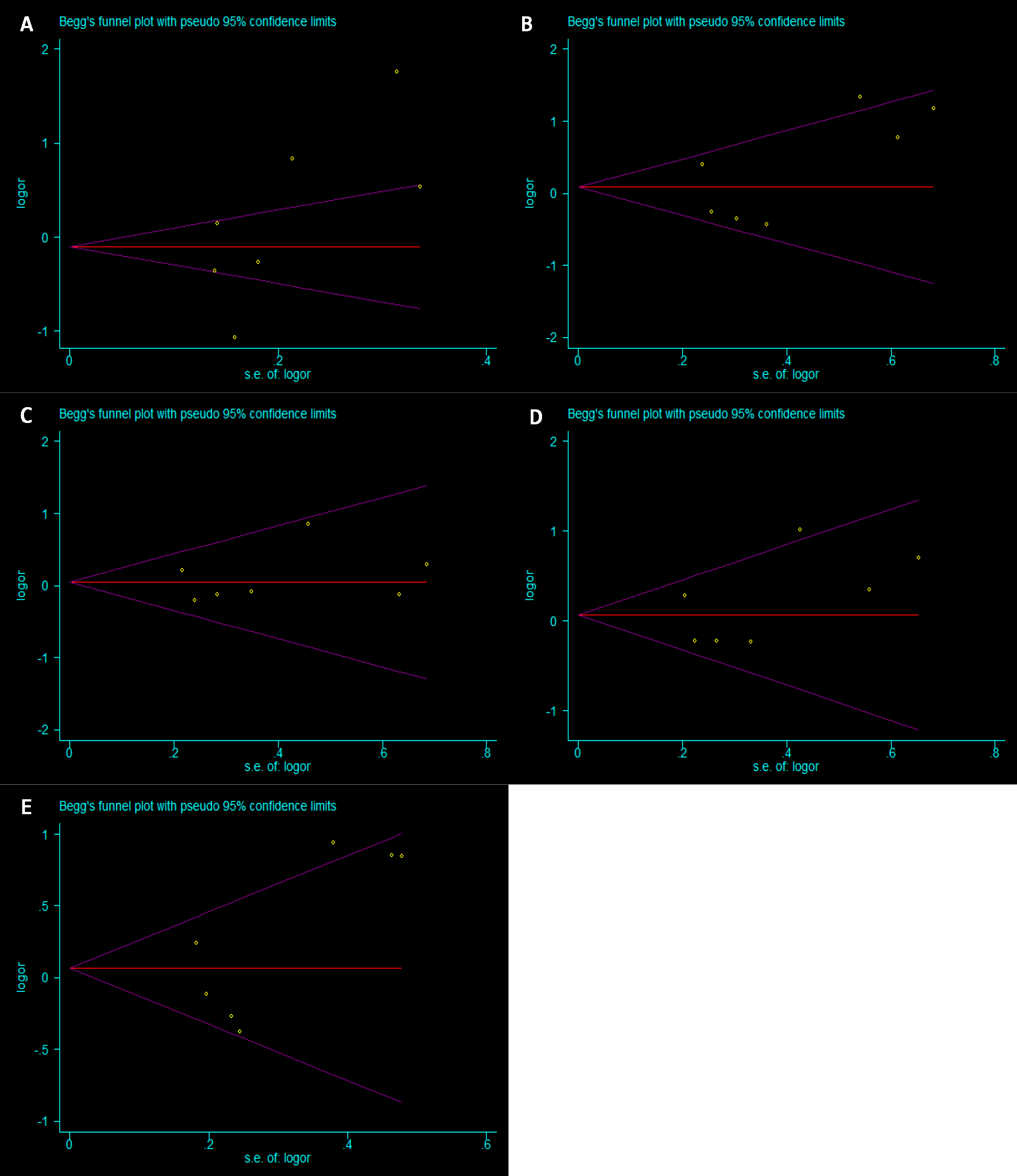

Supplement: Supplementary file 5 — Supplementary Fig. 3. Begg’s funnel plot of bias for studies of the association between SERPINE1 rs1799889 SNP and DR risk. (A) allelic model, (B) homozygote model, (C) heterozygote model, (D) dominant model, and (E) recessive model. [file 12902_2021_837_MOESM5_ESM.tif]

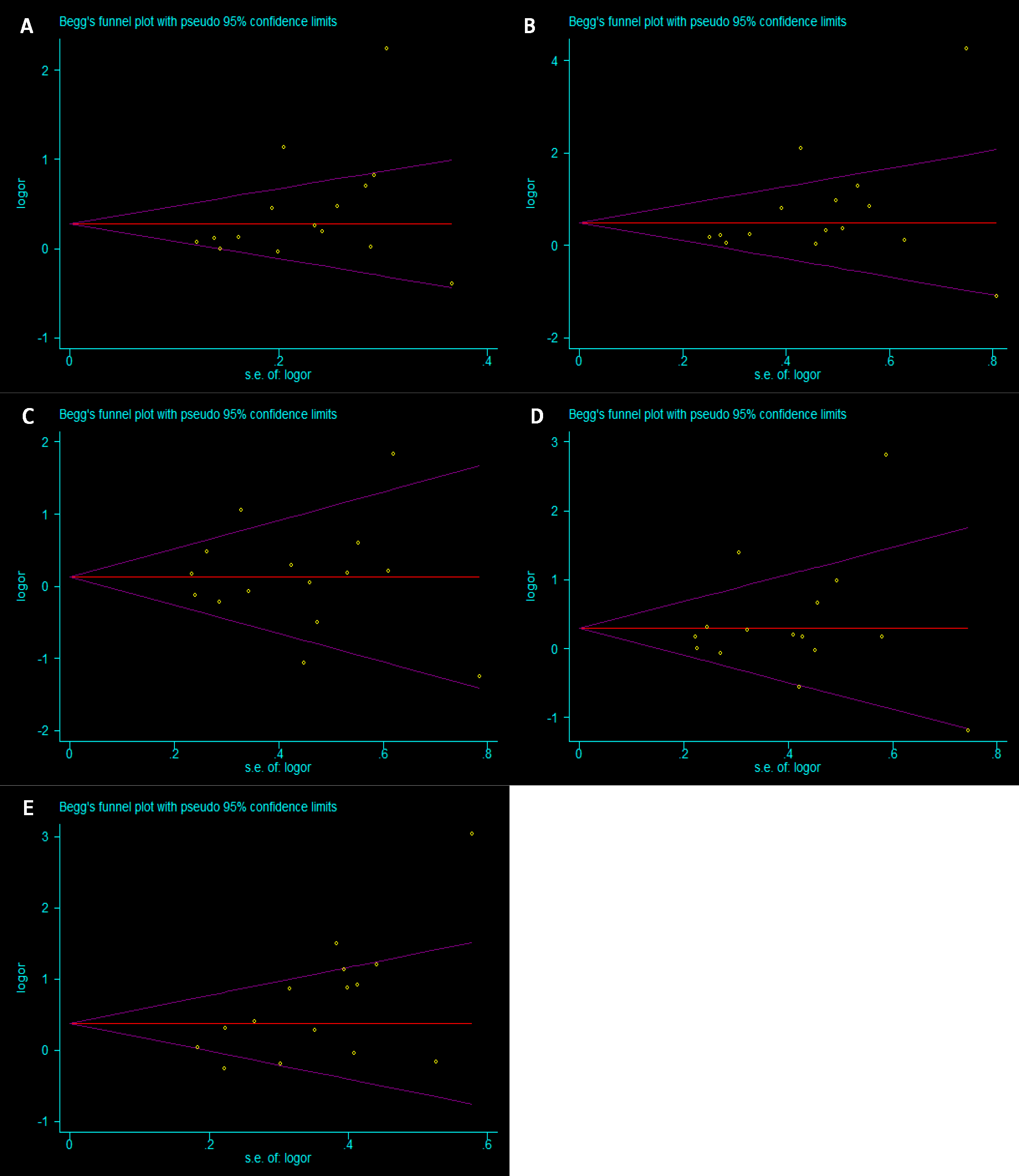

Supplement: Supplementary file 6 — Supplementary Fig. 4. Begg’s funnel plot of bias for studies of the association between SERPINE1 rs1799889 SNP and CVD risk. (A) allelic model, (B) homozygote model, (C) heterozygote model, (D) dominant model, and (E) recessive model. [file 12902_2021_837_MOESM6_ESM.tif]

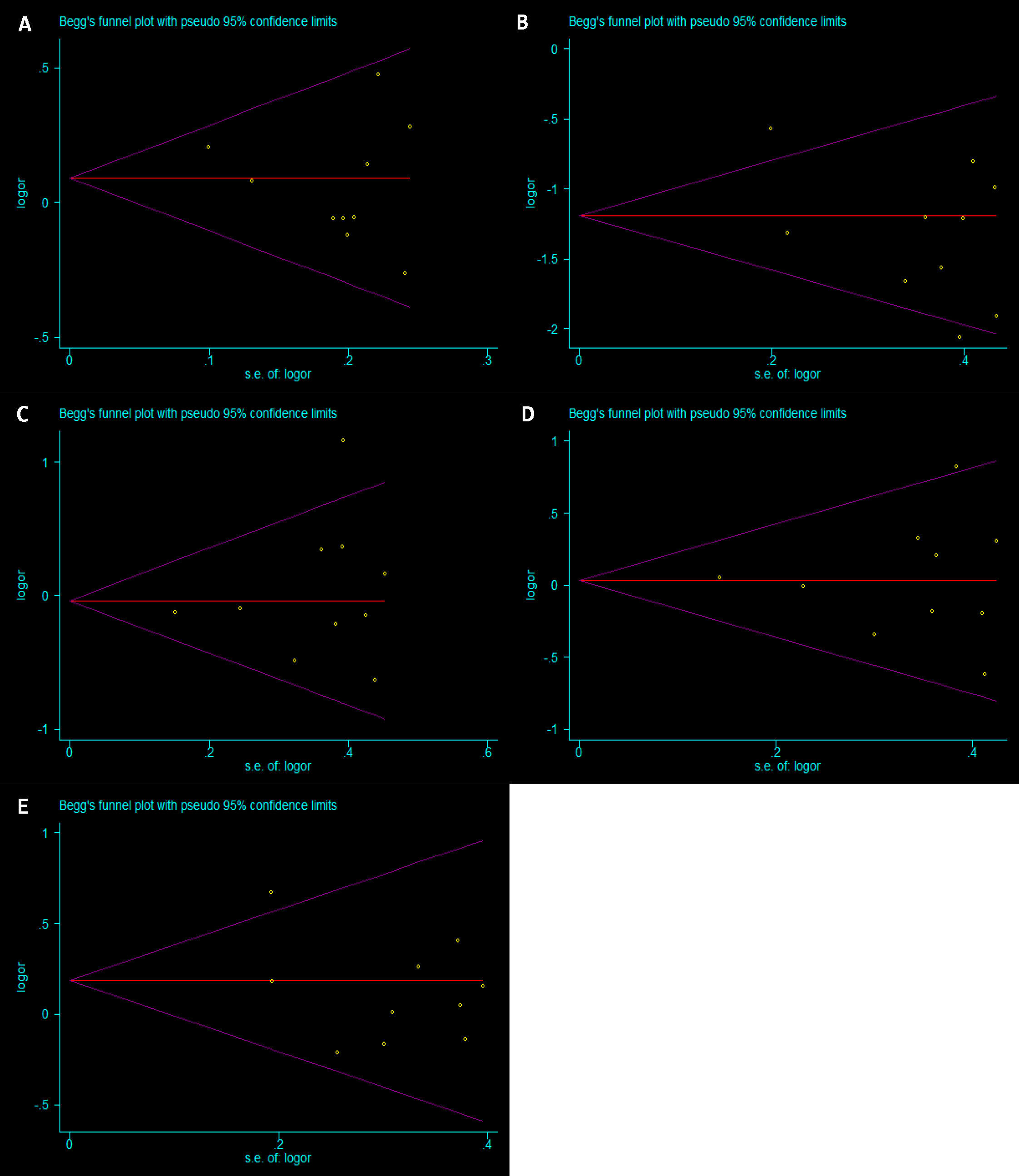

Supplement: Supplementary file 7 — Supplementary Fig. 5. Begg’s funnel plot of bias for studies of the association between SERPINE1 rs1799889 SNP and DN risk. (A) allelic model, (B) homozygote model, (C) heterozygote model, (D) dominant model, and (E) recessive model. [file 12902_2021_837_MOESM7_ESM.tif]
